# Role of Exosomal MicroRNAs and myomiRs in the Development of Cancer Cachexia-Associated Muscle Wasting

**DOI:** 10.3389/fnut.2017.00069

**Published:** 2018-01-09

**Authors:** Rodolfo Marinho, Paulo S. M. Alcântara, José P. Ottoch, Marilia Seelaender

**Affiliations:** ^1^Cancer Metabolism Research Group, Institute of Biomedical Sciences, University of São Paulo, São Paulo, Brazil; ^2^Department of Clinical Surgery, University of São Paulo, São Paulo, Brazil

**Keywords:** exosomes, microRNAs, cachexia, cancer cachexia, inflammation, muscle wasting

## Abstract

Cachexia is a complex metabolic syndrome that promotes great weight loss, with marked muscle mass wasting. In the last years, many efforts have been directed to improve the understanding of the mechanisms involved in the disease. This syndrome is present in up to 80% of cancer patients and, despite its clinical relevance, is underdiagnosed. The orchestration of the molecular and biochemical disruptions observed in cachexia is paralleled by inflammation and the communication among the different body compartments, including the tumor and the skeletal muscle, is still not completely described. One of the mechanisms that may be involved in the transduction of the inflammatory signals and the activation of catabolic status in muscle is the participation of exosomes containing microRNAs (miRNAs) and muscle-specific miRNAs (myomiRs). Exosomes are nanovesicles, measuring from 30 to 100 µm, and able to carry miRNAs in the circulation, promoting cell–cell and tissue–tissue communication in an autocrine, paracrine, and endocrine manner. miRNAs transported in exosomes are preserved from degradation, while these nanoparticles deliver the cargo to specific cell targets, making communication more efficient. Several miRNAs are known to modulate inflammatory pathways, to induce metastasis, to mediate cancer aggressiveness and even to participate in the regulation of protein synthesis and degradation pathways in the skeletal muscle. The aim of this mini-review is to describe the present knowledge about the role of exosomal miRNAs and myomiRs in the induction of muscle mass wasting in cancer cachexia state and to explain which transcription factors, proteins, and pathways are regulated by these molecules.

## Introduction

In the last decade, many efforts were directed to improve understanding of the mechanisms involved in the complex metabolic syndrome of cachexia. This disease is related with marked decrease of body weight and diminished muscle mass, in the presence or absence of fat loss (1, 2). The syndrome is associated with cancer or other chronic inflammatory diseases, affecting quality of life (3–5) and decreasing survival (6, 7). Cancer cachexia affects approximately half of all cancer patients (8); whereas in the more advanced stages of the disease, this estimate may reach 80% (9, 10). In addition, cachexia is considered the immediate cause of death of 20–50% of all cancer patients (5, 6).

Cachectic cancer patients suffer several challenges dealing with the alterations and limitations imposed by both cachexia and chemotherapeutic treatment, which induce fast and pronounced weight loss. Furthermore, cachexia reduces the efficacy and increases the toxicity of chemotherapy (2, 11). Low muscle mass is a predictor for mortality and reflects poor prognosis (2). The cachectic patient may lose up to 75% of his/her skeletal muscle mass (6). Weight loss in cachectic patient is attributable to systemic inflammation, rather than insufficient caloric intake (12).

Despite its clinical relevance, cancer cachexia is underdiagnosed and seldom treated (1, 4). This is probably a reflex of its complexity and of the interaction of factors causing the plethora of symptoms that have been described in the cachectic patient (1, 4, 13). The most prevalent alterations, beyond body mass wasting, are anorexia, fatigue, and impairment of hypothalamic circuits regulating appetite; as well as endocrine disorders and “metabolic chaos,” characterized by marked deregulation of lipid, protein, and carbohydrate metabolism (4, 14).

Despite the complexity of the pathophysiology of cancer cachexia, it has been widely accepted that most alterations are associated with the presence of systemic inflammation. Tan and Fearon (15) proposed five main clusters of symptoms in which inflammation acts as a protagonist in cancer-associated cachexia: (1) systemic inflammation; (2) control of energy balance; (3) function and metabolism of muscles; (4) function and metabolism of the adipose tissue; and (5) modulation of appetite.

The most relevant pro-inflammatory cytokines contributing to the development of cancer cachexia and related with the metabolic alterations leading to muscle mass and adipose tissue wasting are interleukin-1, interleukin-6 (IL-6), tumor necrosis factor alpha (TNF-α), and interferon gamma (5, 7, 16, 17). Peripheral tissues are largely affected by cachexia, even before the detection of anorexia in the patient (18). Hence, loss of muscle mass and fat begins to occur before the patient exhibits a decrease in food intake (18, 19). Increased circulation of the abovementioned cytokines activates lipolysis in the adipose tissue and induces a reduction of protein synthesis, all the while upregulating proteolysis in the muscle (8).

There have been many attempts to describe the mechanisms involved in the onset of inflammation in cancer cachexia; however, such mechanisms are still not fully understood. We have previously shown that the white adipose tissue (WAT) contributes in a robust manner for the increase of circulating inflammatory factors (20–22). More recently, it has been shown (23–26) that WAT actively secretes exosomes containing microRNA (miRNA), which may regulate the inflammatory process in tissues and immune cells. In addition, exosomes from the adipose tissue are able to stimulate and regulate the growth, development, and the aggressiveness of tumors (23, 24).

Many studies demonstrate that tumors are likewise able to secrete exosomes containing miRNAs, which play a role in the activation of the inflammatory process in cancer (27–29). These tumor-derived exosomes interact with mesenchymal stem cells (MSCs) and increase the synthesis and release of pro-inflammatory cytokines, favoring tumor cell survival (27). In addition, exosomes released by the tumor induce the progression of the tumor itself, by modifying tumor microenvironment, and promoting metastasis (27–31).

## myomiRs and Cancer Cachexia

The miRNAs are a family of small, non-coding RNA molecules, composed of 19–24 nucleotides, that regulate gene expression through the degradation of messenger RNA (mRNA) or by inhibiting protein translation (8, 32). The miRNAs were first described in the 1990s, but only in 2002 their involvement in the development of cancer was reported (12). Since the first cancer-related data were obtained, many studies have addressed miRNA participation in important steps of malignant disease, such as in tumor proliferation, apoptosis, migration, and invasion (33–35). In addition, paracrine and/or endocrine actions of miRNA are related with the propagation of systemic inflammation, in the development of metastasis, and in the activation of pathways that promote muscle loss (8).

miRNAs are synthesized from DNA gene transcription by RNA polymerase II, forming primary miRNA (pri-miRNA) transcripts. Subsequently, these pri-miRNAs undergo a process of maturation, when they are cleaved by the Drosha–DGCR8 RNase enzyme complex, generating a double-stranded pre-miRNA (36–38). Drosha is localized in the nucleus and contains two tandem RNase-III domains. After cleavage by Dorsha, the pre-miRNA exhibits an imperfect stem-loop structure with ~70-nucleotides (38). This pre-miRNA is transported from the nucleus to the cytoplasm by exportin5 (XPO5), a Ran-GTP-dependent transporter. In the cytoplasm, miRNAs undergo another maturation step in which the double strand is cleaved by the action of the Dicer enzyme. Dicer cleaves theses hairpin precursors, generating the mature miRNA strand and its complementary strand (miRNA′), these two strands range about 21–25 nucleotides. In sequence, the mature miRNA is bound by Argonaute proteins (Ago) and incorporated into an miRISC effector complex (miRNA induced silencing complex), while the other miRNA strand may be degraded, or still, form another miRISC complex. Alternatively, it may be exported into exosomes to act in a paracrine or endocrine way (37–41).

Changes in the expression profile of miRNAs may indicate the presence and/or the progression of diseases that affect the muscle (7, 37, 42). Alterations such as the upregulation or downregulation of miRNAs have hence been investigated, having provided clear evidence that they can regulate pathways implicated with myogenesis, and skeletal muscle hypertrophy and atrophy (7, 37, 42–44). One of the miRNAs whose concentration is increased, as identified in cancer patients, is miR-21. Several studies have shown an increase of miR-21 in the serum of patients with the most varied types of cancer, such as colorectal cancer (45–47), gastric cancer (48, 49), prostate cancer (50), and hepatocellular carcinoma (51, 52).

Muscle mass loss during cachexia is related with an increase in protein degradation and metabolic changes in the muscle, in response to the presence of the tumor in the organism (42). Soares et al. (43) observed different profiles of alterations in miRNA expression with *in vitro* and *in vivo* approaches in four different animal models of skeletal muscle wasting: starvation, denervation, diabetes, and cancer cachexia. The authors identified that, following the denervation protocol, miR-206 and miR-21 are upregulated and promote muscle atrophy. These two miRNAs are capable of binding to the transcription factor YY1, as well as the translational initiation factor eIF4E3, and regulate muscle mass wasting, thus interfering in myogenesis (43).

In addition, other pathways involving miRNAs and the regulation of myogenesis, hypertrophy, or atrophy have been described both *in vitro* and *in vivo*. Koutalianos et al. (53) showed that overexpression of MyoD, a myogenic transcription factor, induces the expression of miR-206. In addition, the increase of miR-206 downregulates Twist-1, decreasing the activity of Twist-1, and allowing increased differentiation of muscle cells. The authors reported that muscle cells from patients with myotonic dystrophy type 1 exhibited inhibition of MyoD protein expression and an increase of Twist-1 expression, following a reduction in miR-206 levels. The co-transfection of MyoD and miR-206 regulated the protein content of Twist-1, allowing the differentiation of muscle cells to occur (53).

Moreover, Kukreti et al. (54) showed that dexamethasone or myostatin induces atrophy of skeletal muscle through miR-1 expression modulation. The authors demonstrated that miR-1 can bind to and reduce heat shock protein 70 (HSP70) action, thus participating in the induction of atrophy. Decreased levels of HSP70 are associated with the downregulation of Akt phosphorylation (p-Akt), since HSP70 binds to and protects the integrity of p-Akt. The decrease in p-Akt promotes a reduction of Foxo3 phosphorylation, allowing for enhanced nuclear activity of Foxo3, and promoting upregulation of muscle finger protein (MuRF1) and atrogin-1; both of which induce the atrophy program in the skeletal muscle (54).

Remarkably, Narasimhan et al. (7), when analyzing *rectus abdominis* biopsies from cancer and cachectic cancer patients, employing next-generation sequencing, discovered eight miRNAs that are upregulated in cancer-associated cachexia and associated with muscle metabolism, myogenesis, and inflammation. The upregulated miRNAs are as follows: hsa-let-7d-3p; hsa-miR-345-5p; hsa-miR-423-5p; hsa-miR-532-5p; hsa-miR-1296-5p; hsa-miR-3184-3p; hsa-miR-423-3p; and hsa-miR-199a-3p (7).

The authors (7) described that: (1) let-7d-3p is related with transferrin receptor, promoting downregulation of this pathway, affecting muscle cell proliferation and myogenic differentiation; (2) miR-345-5p has NOV and COL1A1 genes as targets, downregulating these and upregulating CYR61. NOV and CYR61 are involved in insulin-like growth factor 1, Akt and mTOR pathways, reducing the capacity to protein synthesis; (3) miR-423-5p and miR-3184-3p downregulate two genes, SQLE and FADS2. These genes are related with lipid biosynthesis; miR-423-5p also regulates leptin and other genes associated with energy balance; in addition, miR-423-5p downregulates DLK1, which is involved with muscle hypertrophy; while (4) miR-423-3p promotes a reduction of calcium signaling, affecting CAMK2A gene; (5) miR-3184-3p is involved with Wnt/β-catenin signaling, impairing myogenic differentiation. In addition, miR-3184-3p regulates BMPR1B and GREM1, and this way, transforming growth factor β and BMP signaling are affected; (6) miR-532-5p interferes with SULF1, RPS6KA6, and NPY1R genes. SULF1 is related with BMP signaling, influencing somite development; NPY1R is involved with appetite regulation, and RPSKA6 participates in ciliary neurotrophic factor (CNTF) actions; (7) miR-1296-5p regulates HTR2A and RPS6KA6 genes. HTR2A participates in serotonin signaling (serotonin is involving in myogenesis), while RPS6KA6 is involved with CNTF signaling; and (8) miR-199a-3p affects the EIF4EBP1 gene. This gene regulates the mTOR pathway, interfering in protein synthesis (7).

**Figure 1:**
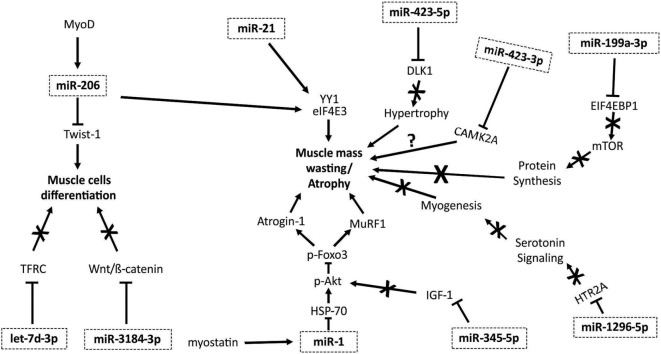
myomiRs and muscle mass wasting and atrophy pathways. MyoD is able to activate miR-206, promoting an inhibiting effect on Twist-1 protein, diminishing muscle cell differentiation (53). miR-206 and miR-21 regulate the action of YY1 and eIF4E3, promoting muscle mass wasting (43). Myostatin increases miR-1 expression, promoting a reduction in heat shock protein 70 (HSP70) action, decreasing Akt phosphorylation, and its regulation of Foxo3. When Foxo3 is not phosphorylated, the expression of several proteins that induce atrophy, including Atrogin-1 and MuRF1, are enhanced (54). The let-7d-3p downregulates transferrin receptor (TFRC), affecting muscle cell proliferation and myogenic differentiation (7). miR-3184-3p inhibits Wnt/β-catenin pathway, impairing myogenic differentiation (7). mir-345-5p downregulates genes and proteins involved in IGF1 pathway, decreasing anabolic signaling. miR-1296-5p regulates HTR2A gene, reducing the participation of serotonin in the induction of myogenesis (7). miR-199a-3p affects the EIF4EBP1 gene, reducing mTOR pathway activity, interfering in protein synthesis (7). miR-423-3p promotes reduction of calcium signaling, affecting CAMK2A gene (7). miR-423-5p downregulates DLK1, what is involved with muscle hypertrophy; thus, reducing this capacity (7).

Taken together, these recently published findings demonstrate the role of myomiRs in the modulation of atrophic pathways, providing insight on the possible relevant role of these molecules in cancer cachexia. Figure 1 summarizes the mechanisms by which myomiRs modulate atrophy and muscle mass wasting.

## Exosomal miRNAs and Muscle Wasting in Cancer Cachexia

Exosomes are small membrane-derived particles, ranging from about 30 to 100 µm (42). The biogenesis of exosomes is linked to the synthesis of miRNAs (8, 55). Due to high stability and specificity for delivering the cargo to the target cells, exosomes are involved in tissue-tissue communication in an autocrine, paracrine, and endocrine way (55, 56). These particles have been shown to represent an efficient way to transport other molecules, such as proteins, some types of RNAs (intact mRNA; mRNA fragments; long non-coding RNA; miRNA; ribosomal RNA; fragments of tRNA), and cytokines, reducing degradation due to transport in bloodstream (28, 55, 57).

The initiation and maintenance of cachexia-related inflammation may present a major contribution of miRNAs (7, 8, 12, 32). These actions are associated with the presence of miRNA-enriched circulating exosomes (8, 42). He et al. (32) demonstrated a mechanism underlying the participation of exosomal miRNAs in muscle mass wasting. The authors reported that lung and pancreatic cancer cells secrete exosomes containing miR-21. These are transported in the bloodstream and induce apoptosis of muscle cells. This phenomenon happens because miR-21 is able to bind and activate toll-like receptor 7 (TLR-7) in rat cells and toll-like receptor 8 (TLR-8) in human myoblasts, thereby promoting apoptosis, through the activation of the c-Jun N-terminal kinase pathway (JNK) (32).

In another study, Hudson and colleagues (58) demonstrated that miR-182 present in isolated exosomes could attenuate the role of Foxo3 in inducing atrophy in the skeletal muscle. Foxo3 is a transcript factor that promotes the increase of atrophy-related genes such as atrogin-1, autophagy-related protein 12 (ATG12), and microtubule-associated protein light chain 3. The authors demonstrated that treatment of C2C12 myotubes with dexamethasone promotes a reduction of the expression of miR-182 and an increase in autophagy, *via* enhanced activity of Foxo3. The same atrophy program was observed in the gastrocnemius muscle of diabetic (induced by streptozotocin) rats, with diminished expression of miR-182, and enhanced Foxo3 mRNA (58).

**Figure 2:**
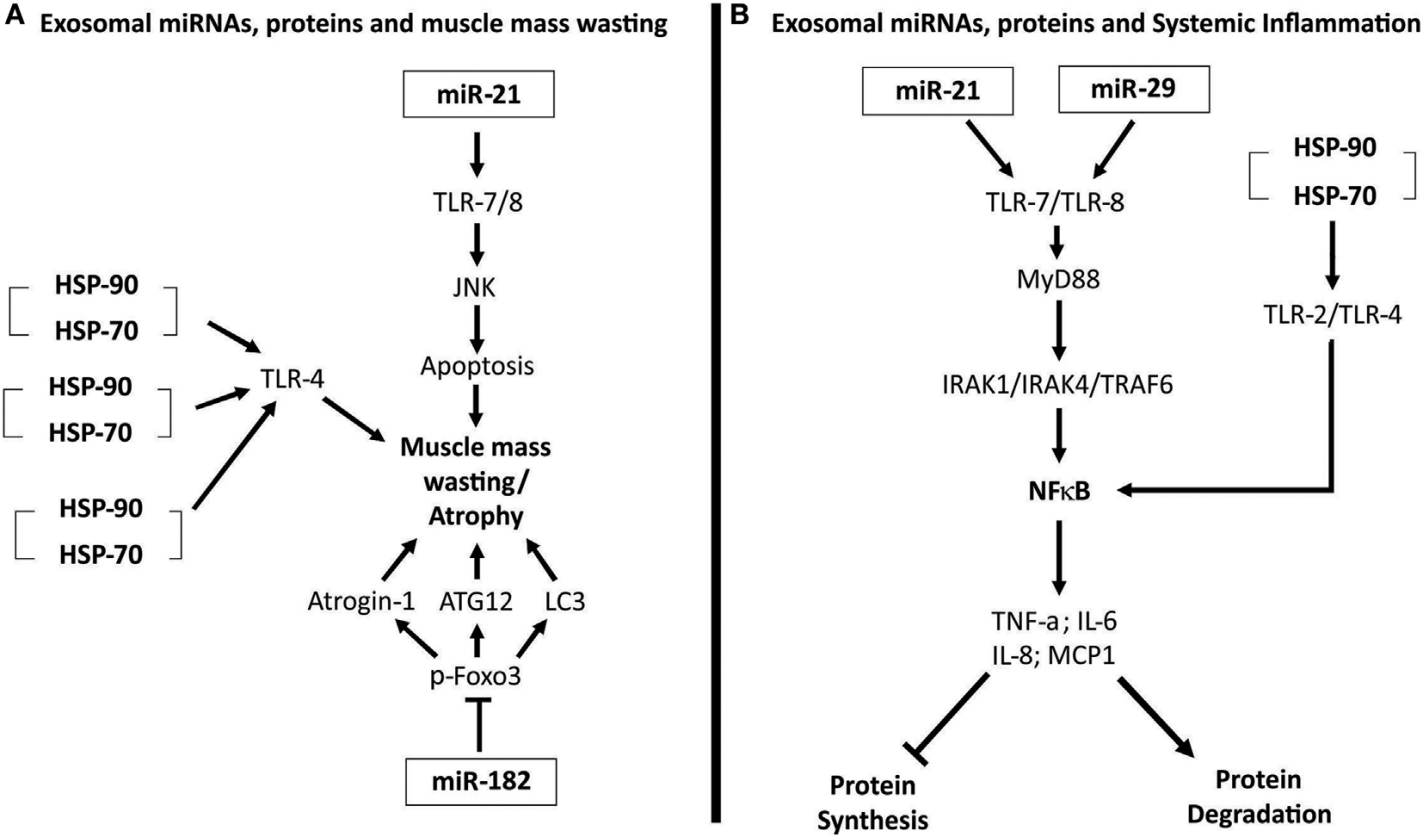
**(A)** Exosomal microRNAs (miRNAs), proteins, and muscle mass wasting. miR-21 interacts with and activates toll-like receptor 7 (TLR-7). TLR-7, through the c-Jun N-terminal kinase pathway (JNK) pathway, induces apoptosis of muscle cells, leading to atrophy (32). Heat shock protein 70 (HSP70) and HSP90 that compose the membrane of exosomes can bind to TLR-4, activating this receptor on muscle cells and induce the muscle mass wasting (11). Exosomal miR-182 is able to block transcript factor Foxo3’s action, inhibiting its action, reducing the expression of several atrophy genes, as light chain 3 (LC3), Atrogin-1, and ATG12 (58). **(B)** Exosomal miRNAs, proteins, and systemic inflammation. miR-21 and miR-29 are able to interact and activate TLR-7/8–MyD88–nuclear factor kappa B (NFκB) pathway, inducing an increased expression and release of pro-inflammatory cytokines tumor necrosis factor alpha (TNF-α), interleukin-6 (IL-6), IL-8, and MCP-1 (59, 60). This pro-inflammatory state enhances protein degradation and inhibits protein synthesis. HSP70 and HSP90 present in exosomes membranes can bind to, and activate TLR-2 and TLR-4. When activated, these receptors promote the activation of the NFκB pathway, inducing a pro-inflammatory status (11).

Recently, an elegant study performed by Zhang et al. (11) demonstrated that cancer cells release exosomes and that HSP70 and HSP90 proteins in the membrane of exosomes induce muscle mass wasting in cancer cachexia models. Extracellular HSP70 and HSP90 function as danger-associated molecular patterns (11). These two proteins can interact with TLR-4 and promote its activation, and thus, activate p38β MAPK-C/EBPβ catabolic signaling pathway in the muscle (11). Figure 2A illustrates the mechanisms by which exosomal miRNAs and proteins induce muscle mass wasting in cancer cachexia.

## Exosomal miRNAs and Systemic Inflammation

In parallel to the involvement of miRNAs in the regulation of muscle wasting and atrophy pathways, recent studies have shown that exosomes contain miRNAs and are also able to promote and perpetuate systemic inflammation present in cachectic cancer state (8, 59–62). Fabbri and colleagues demonstrated that miRNAs 21 and 29 are able to associate and activate TLR-7 and TLR-8, triggering an inflammatory signal. When activated, toll-like receptors promote the recruitment of MyD88 and the formation of the complex IRAK1, IRAK4, and TRAF6, which then activates nuclear factor kappa B (NFκB). In the nucleus, NFκB induces pro-inflammatory cytokines expression, including TNF-α and IL-6 (59, 60).

Furthermore, Li et al. (61) reported that when MSCs were incubated with exosomes derived from lung tumor cell lineage A549, the presence of those vesicles induces a pro-inflammatory phenotype in MSCs. These cells enhance the synthesis and secretion of IL-6, IL-8, and MCP-1. The authors further demonstrated that HSP70 present in the membrane of exosomes is able to bind to TLR-2 and then activate the NFκB pathway, leading to increased expression and secretion of the inflammatory cytokines IL-6, IL-8, and MCP-1 (61).

In addition, Zhang et al. (11) showed that HSP70 and HSP90 proteins, existing in exosomes membrane, interact and activate TLR-2 and TLR-4 on immune cells, triggering innate immune response. The activation of these receptors promotes an increase in the synthesis and release of pro-inflammatory cytokines (TNF-α and IL-6), collaborating to the development of systemic inflammation existing in cancer cachexia (11). Figure 2B illustrates the role of exosomal miRNAs and proteins in the perpetuation of systemic inflammation.

Collectively, these findings demonstrate an important involvement of exosomal miRNAs and proteins for the activation and perpetuation of systemic inflammation in cancer and in the induction of cancer-associated cachexia. One such systemic inflammation scenario exerts a negative influence on muscle metabolism, regulating signaling pathways involved in the synthesis and breakdown of proteins in muscle, collaborating with the development of muscle mass wasting in cancer cachexia (8, 11, 17, 59, 60, 63).

Despite the great potential in the study about exosomes, miRNAs and exosomal miRNAs, further studies are needed to enhance understanding of details in regard to some processes: steps of exosome biogenesis are not yet fully known; the mechanisms underlying sorting of each miRNA into exosome particles are likewise, not totally elucidated. In addition, it remains to be established what pathways are regulated by each miRNA; what and which functions do the proteins present in the exosomes membrane exert (28). Moreover, the development of more precise and refined methods for exosome isolation are needed, as well as improvements in protein detection techniques, allowing improved characterization of each exosome and particular functions in the pathophysiology of cachexia (55, 56, 64).

## Conclusion

The study of exosomal miRNAs and myomiRs is a promising field of research for improving the understanding of cancer cachexia mechanisms. Both, exosomal miRNAs and myomiRs participate directly and/or indirectly in muscle mass wasting, accentuating protein degradation pathways and inhibiting myogenesis. Of particular interest, are exosomal-transported miRNAs as they seem to be markedly involved in the development and perpetuation of inflammatory status in cancer cachexia.

The role of exosomes in cancer cachexia is thus of great interest, as these nanovesicles have the capacity to facilitate communication among several tissues in a paracrine and endocrine manner, by carrying proteins and miRNAs. As cachexia is a syndrome with systemic effects in which tissue cross talk is prominent, these particles pose as likely candidates to intermediate the changes, and therefore, improved characterization and knowledge about the biogenesis and functions of exosomes and exosomal miRNAs, may promote the use of these extracellular vesicles as follows: (1) biomarkers, in the quest for faster and more accurate diagnosis, or for monitoring the evolution of the disease and (2) in the development of more specific antitumor drugs, which could diminish the release of inflammatory factors and/or factors associated with muscle mass wasting in cachexia.

## Author Contributions

All the authors contributed equally to this work. All the authors have read and approved the final manuscript.

## Conflict of Interest Statement

The authors declare that the research was conducted in the absence of any commercial or financial relationships that could be construed as a potential conflict of interest.
